# Total and Envelope Protein-Specific Antibody-Secreting Cell Response in Pediatric Dengue Is Highly Modulated by Age and Subsequent Infections

**DOI:** 10.1371/journal.pone.0161795

**Published:** 2016-08-25

**Authors:** Jessica F. Toro, Doris M. Salgado, Rocío Vega, Jairo A. Rodríguez, Luz-Stella Rodríguez, Juana Angel, Manuel A. Franco, Harry B. Greenberg, Carlos F. Narváez

**Affiliations:** 1 Programa de Medicina, Facultad de Salud, Universidad Surcolombiana, Neiva, Colombia; 2 Departamento de Pediatría, Hospital Universitario de Neiva, Neiva, Colombia; 3 Instituto de Genética Humana, Pontificia Universidad Javeriana, Bogotá, Colombia; 4 Departments of Medicine and Microbiology & Immunology, Stanford University School of Medicine, Stanford, California, United States of America; Institut Pasteur of Shanghai Chinese Academy of Sciences, CHINA

## Abstract

The response of antibody-secreting cells (ASC) induced by dengue has only recently started to be characterized. We propose that young age and previous infections could be simple factors that affect this response. Here, we evaluated the primary and secondary responses of circulating ASC in infants (6–12 months old) and children (1–14 years old) infected with dengue showing different degrees of clinical severity. The ASC response was delayed and of lower magnitude in infants, compared with older children. In primary infection (PI), the total and envelope (E) protein-specific IgM ASC were dominant in infants but not in children, and a negative correlation was found between age and the number of IgM ASC (rho = −0.59, P = 0.03). However, infants with plasma dengue-specific IgG detectable in the acute phase developed an intense ASC response largely dominated by IgG and comparable to that of children with secondary infection (SI). IgM and IgG produced by ASC circulating in PI or SI were highly cross-reactive among the four serotypes. Dengue infection caused the disturbance of B cell subsets, particularly a decrease in the relative frequency of naïve B cells. Higher frequencies of total and E protein-specific IgM ASC in the infants and IgG in the children were associated with clinically severe forms of infection. Therefore, the ASC response induced by dengue is highly influenced by the age at which infection occurs and previous immune status, and its magnitude is a relevant element in the clinical outcome. These results are important in the search for correlates of protection and for determining the ideal age for vaccinating against dengue.

## Introduction

Dengue is an acute febrile viral disease with a high burden worldwide. The dengue virus (DENV) has been estimated to originate 390 million infections annually, 100 million of them symptomatic [1]. The disease is caused by infection with one of four interrelated DENV serotypes (DENV-1 to DENV-4), which use mosquitoes of the *Aedes* genus as a vector. Clinically, dengue has a range of manifestations, which vary from asymptomatic profiles or mild disease to potentially lethal conditions, characterized by shock, hemorrhages or organ failures [2]. Recently, the WHO recommended using a revised classification for dengue in which clinical parameters are the main factors. This classification has proven useful in the early identification of severe cases of dengue, although its utility in physiopathological studies is still disputed [3].

Neutralizing antibodies are a main protective factor against DENV. Infection with one serotype provides long-term homotypic and heterotypic protection for a few months immediately following the infectious event [4]. Neutralizing antibodies recognize epitopes particularly on the envelope (E) protein, a viral glycoprotein that mediates adhesion to cellular receptors, although most serum antibodies against E protein are cross-reactive between four DENV serotypes [5]. In addition to E protein, nonstructural 1 (NS1) and premembrane (prM) proteins are other immunodominant viral proteins that are targets of human antibodies [6].

Most clinically relevant DENV infections occur in individuals with preexisting virus-specific antibodies [7]. In antibody-dependent enhancement, the existence of non-neutralizing or sub-neutralizing heterotypic antibodies generated by previous infection increases the attraction of viral particles to immune cells, which express Fcγ receptors, amplifying infection and the activation and secretion of inflammatory mediators [8]. Thus, humoral immunity to DENV has also been shown to play a critical role in dengue pathogenesis.

Although serum antibodies have been extensively studied, antibody-secreting cells (ASC) induced by viral infection have only recently started to be characterized. Of the studies conducted in adults and older children with secondary infection (SI), it is known that infection with DENV induces a strong ASC response in the acute phase. Dengue-specific IgG ASC that cross-reacts among the four serotypes massively dominates the response, and the virus-specific plasma IgG titer has been shown to partially correlate with the DENV-specific ASC frequencies detected by ELISPOT [9–11]. In contrast to SI, many aspects of the ASC response in primary infection (PI), such as the expressed isotype, magnitude and association with clinical manifestations, are still unclear. Due to the existence of amplifying maternal antibodies transferred through the placenta, a significant fraction of symptomatic PIs are established in infants [12]. Classically, differences have been noted in the immunity of infants and children to pathogens, including a hindered Th-1 response, a low production capacity of IFN type 1 and a low frequency of T and B memory lymphocytes due to limited antigenic exposure in early life, among others [13]. In dengue, for example, monocytes from neonates treated *in vitro* with the virus when compared with monocytes from adults, produce significantly lower amounts of TNF-α and IL-6 [14]. We hypothesized that the ASC response induced by natural infection with DENV is determined by two simple factors: age and previous infections. Therefore, we analyzed the total and E protein-specific ASC response in young patients with different clinical stages of dengue; these patients included 6- to 12-month-old infants and 13 to 168 months old children (1- to 14-year-old). Our findings show that the ASC response in the infants was qualitatively and quantitatively different from that of the older children. These differences were consistent with the respective titers of plasma antibodies. Differences were found in circulating ASC between PIs and SIs. Depending on age, the intensity of the ASC response was associated with the clinical characteristics of the infection.

## Materials and Methods

### Ethics statement

This study was approved by the ethics committees of the Facultad de Salud Universidad Surcolombiana (Approval code: NCS-046) and the Hospital Universitario de Neiva (Approval code: 12-22-031). Informed consent was signed by the parents or guardians of the children. This study was conducted according to the human research policies of Universidad Surcolombiana and the Hospital Universitario de Neiva and followed all the statements expressed in the Helsinki Agreement.

### Patients and samples

In this study, performed from June 2011 to July 2013 in a dengue endemic area, 116 children with dengue admitted to ESE Carmen Emilia Ospina (a primary health care center) and at the Hospital Universitario de Neiva (a tertiary hospital) were included. Forty healthy children were invited to participate as controls in the Facultad de Salud, Universidad Surcolombiana. For the healthy children, a medical exam and complete blood count was realized by the Pediatric Division, Hospital Universitario de Neiva. For the children with dengue, the enrollment criteria included an age between 6 months and 14 years and fever of fewer than 6 days evolution, accompanied by headache, retroocular pain, myalgia, arthralgia, rash, and no obvious source of infection. Thirty-five of the children were included from 6- to 12-month-old infants. The children with dengue were classified into dengue with or without warning signs (DWS or DNS, respectively) and severe dengue (SD) according to the revised WHO 2009 guidelines [15]. Children with DWS and those with SD were admitted to the Service of Pediatrics and the Pediatric Intensive Care Unit of the Hospital Universitario de Neiva, respectively, where clinical and daily laboratory monitoring was performed, including procedures such as blood cell count, coagulation times, liver enzymes, chest X-rays and electrocardiograms, among others.

Two 2- to 4-ml samples of peripheral blood (weight adjusted) were taken, one during the acute phase (the day of admission) and one during the convalescent phase (≥20 days since fever onset). For analysis of the dynamics of circulating ASC, a consecutive daily sample was taken from a fraction of the children during hospitalization from routine control blood samples. Samples were centrifuged at 200x*g* and plasmas were collected and preserved at -70°C until used. Peripheral blood mononuclear cells (PBMCs) were isolated, as described below, within 4 h of the time of collection. All cellular experiments including the ELISPOT and B cells phenotypic analysis were realized on fresh PBMCs within 6h after bleeding.

### Diagnosis of dengue and detection of PI or SI

Any case that met two of the following three conditions was taken as a confirmed case of dengue: 1). Detection of viral genome by reverse transcription polymerase chain reaction (RT-PCR), 2.) NS1 detection or 3). DENV-specific IgM, all evaluated in plasma. For the detection of viral genome and infecting serotype a RT-PCR assay was applied, as previously reported [16].

Detections of NS1, DENV-specific IgM and IgG in plasma were performed by ELISA during the acute phase with commercial kits (Dengue Early, catalog no: E-DEN02-P, Dengue IgM Capture, catalog no: 01PE20 and Dengue IgG Capture, catalog no: 01PE10, respectively), following all manufacturers’ instructions. In certain experiments, the titers of DENV-specific IgM in plasma were determined with the respective kit using serial dilutions.

Infants and children with the presence of viral RNA and/or NS1 and/or IgM and the absence of DENV-specific IgG in plasma taken during the acute phase were classified as having a PI. Also, children with a plasma DENV-specific-IgM / IgG ratio ≥2.0 or <2 in acute-phase were catalogued as having a PI or SI, respectively, as previously reported [17]. Because of potential maternal IgG, infants with plasma DENV-specific IgG were not classified as SI (n = 7).

### Isolation of PBMCs

PBMCs were isolated by Ficoll density gradient centrifugation (Ficoll-Paque^™^ Plus, catalog no: 17-14440-02, GE Healthcare, Uppsala, SW). Then, cells were washed twice with RPMI 1640 supplemented with 10% of fetal bovine serum (FBS), 2mM L-glutamine, 100 U/ml penicillin, 100 μg/ml streptomicyn, 0.1 mM non-essential aminoacid, 1 mM sodic pyruvate and 0.05 mM β-mercaptoethanol ([Complete medium], all reagents obtained from GIBCO, NY, USA). Finally, the isolated PBMCs were counted by exclusion staining with trypan blue at 0.4% (Catalog no: 145–0013, BIO-RAD, UK).

### B cells phenotype and intracellular expression of immunoglobulin

Antibody-secreting cells were detected by flow cytometry (FC) as previously reported [18]. A total of 5x10^5^ PBMCs were washed with staining buffer (0.5% bovine serum albumin [BSA] [catalog no. A7906; Sigma-Aldrich, St. Louis, MO] and 0.02% sodium azide [catalog no. 106688; Merck, Darmstadt, Germany] in phosphate-buffered saline [PBS], filtered). Subsequently, optimized concentrations of anti-human CD19 APC (clone HIB19), CD20 APC-Cy7 (clone L27), IgD FITC (clone IA6-2), CD27 PE (clone M-T271) and CD38 PerCP-Cy5.5 (clone HIT2) all from Becton Dickinson, were added. After 30 min of incubation at 4°C, the cells were washed with staining buffer and finally fixed with 300 μl of 1% paraformaldehyde (Catalog no: 15712-S, Electron Microscopy Sciences, Hatfield, PA).

The intracellular expression of Igs in B cells (Bc) was determined in 33 children with dengue. For this, 1x10^6^ PBMCs were washed and stained with the previously mentioned antibodies, except for anti-CD19 APC, which was replaced by anti-CD19 PE-Cy7. The cells were incubated for 30 min at 4°C protected from light. After a wash with staining buffer, 300 μl of Cytofix/Cytoperm (Catalog no: 554722, Becton Dickinson, San Jose, CA, USA) was added. After 20 min of incubation at 4°C, the cells were washed twice with 700 μl of Perm/Wash buffer (Catalog no: 554723, BD) and stained intracellularly with F(ab´)_2_ fragment donkey anti-human IgM-APC (Catalog no: 709-136-076, Jackson ImmunoResearch, West Grove, PA) and anti-IgG V450 (Catalog no: 361299, clone G18-145, BD) for 30 min at 4°C. After two washes with Perm/Wash, the cells were finally resuspended in 300 μl of Perm/Wash solution. All the samples were acquired within 1 h of stopping the staining, using DIVA software v.6 1.3 and a FACS Canto II flow cytometer (BD). At least 20,000 CD19^+^ events were acquired in all cases. In a fraction of experiments fluorescent minus one controls were introduced.

### Total and antigen-specific two-color enzyme-linked immunospot (ELISPOT)

To determine the frequency of ASC producing total (non antigen-specific) and E protein-specific Igs, a two-color ELISPOT assay was used as previously described [19]. E protein was selected because most of serum antibodies are directed toward this protein after a DENV infection [6]. Clear, non-sterile, 96-well MultiScreen HTS plates (Catalog no: MSIPN4510, Millipore, Billerica, MA, USA) were covered with 70 μl of affinity-purified goat anti-human IgA+IgM+IgG (H+L) (Catalog no: 01-10-17, KPL, Gaithersburg, MD), at a concentration of 4 μg/ml in sterile PBS or with a mixture of ≥95% pure recombinant E protein from each of the four serotypes generated in insect cells (Hawaii Biotec Inc., Aiea, HI), and used at a concentration of 100 μg/ml. This concentration was shown to have the best signal to noise ratio and the largest number of defined spots in preliminary titration experiments performed on the PBMCs of children with ongoing DENV infection (n = 3, data not shown). Wells covered with sterile PBS were used as a negative control. The plates were covered and incubated at 4°C overnight and were blocked with 200 μl/well of complete medium for 2 h before use. Fresh PBMCs (obtained less than 6 h from sample collection) were resuspended in complete medium that contained 0.5 μg/ml Reserve AP goat anti-human IgG phosphatase-labeled H+L (Catalog no: 4751–1006, KPL, Gaithersburg, MD) and 5 μg/ml of goat anti-human IgM peroxidase conjugate (Catalog no: A6907, Sigma, St Louis, MO). For the detection of total IgA ASC, PBMCs were resuspended in complete medium that contained goat anti-human IgA peroxidase conjugate (Catalog no: A0295, Sigma, St Louis, MO) at a concentration of 6.3 μg/ml and placed in separate wells. Eight serial dilutions were performed starting at 100,000 and 200,000 to detect total and E protein-specific ASC, respectively. The plates were incubated at 37°C in 5% CO_2_ for 4 h and then washed three times with sterile 1X PBS and revealed first with an aminoethylcarbazole (AEC)-peroxidase substrate kit and subsequently with an alkaline phosphatase substrate kit III (Catalogs: SK-4200 and SK5300, respectively, VECTOR, Burlingame, CA, USA), according to manufacturer’s instructions. IgG and IgM ASC were visualized as blue and red spots in the same well, respectively. Two experienced individuals counted the spots with a dissection microscope, and the mean of the two counts are reported. In all experiments, the background given by the wells covered with PBS was subtracted from the final count. Finally, the result was reported as the number of total and E protein-specific ASC / 10^6^ PBMCs.

### Evaluation of E protein-specific IgG in plasma

The relative amount of E protein-specific IgG in plasma was evaluated with an in-house ELISA. MaxiSorp^™^ 96-well plates (Nunc^™^, Denmark), were covered overnight at 4°C with 100 ng/well of a mixture of recombinant E protein of the four serotypes diluted in sterile PBS. The plates were blocked for 1 h at 37°C with 5% non-fat dry milk and 0.1% Tween 20 (Catalog no: 23336–2500, ACROS, NJ, USA) in sterile PBS (5% Blotto); plasma samples diluted in 2.5% Blotto (70 μl/well) were then placed in serial dilutions, starting with 1/100 in duplicates, and were incubated for 2 h at 37°C. Subsequently, the plates were washed with wash buffer (Tween 20, 0.1% in PBS), and 70 μl/well of goat anti-human IgG biotin labeled (Catalog no: 16-10-02, KPL) diluted in 2.5% Blotto at a concentration of 0.5 μg/ml was added and incubated for 1 h at 37°C. The plates were washed with wash buffer, and 70 μl/well of streptavidin-peroxidase (Catalog no: 14-30-00, KPL) diluted in 2.5% Blotto at a concentration of 0.5 μg/ml was added and incubated for 1 h at 37°C. After four washes with wash buffer, 70 μl/well of tetramethylbezidine substrate system (Catalog no: 50-76-03, KPL) was added. The reaction was stopped with 17.5 μl/well of 2 M sulfuric acid. The plate was read in a Multiskan FC microplate photometer (Thermo Scientific) at 450 nm. In all experiments, a pool of plasma from children previously classified as DENV-IgG seropositive or seronegative for ELISA capture (Panbio, Alere, AUS) were used as controls. In addition, wells covered with PBS were also included to determine the background of the assay.

### Statistical analysis

The GraphPad Prism 6.0 (GraphPad Software, La Jolla, CA) was used for the statistical analysis. Since data were not normally distributed as shown with a Shapiro-Wilk test, nonparametric test were used. To compare two independent or paired groups, the Mann-Whitney and Wilcoxon tests were used, respectively. The Kruskal-Wallis test was selected when three or more independent groups were compared. If Kruskal-Wallis test P was lower than 0.05, Dunn´s multiple comparison test was used as post hoc test. To establish the correlation between two variables, the Spearman rank test was selected. When a frequency analysis was required the two-tailed exact Fisher´s test was used. Differences were considered significant if P<0.05. The median and range are shown for all data.

## Results

### Characteristics of the included patients

In this study, 40 healthy subjects, and 49 with PI and 60 with SI were analyzed. The participants were divided into infants (6 to 12 months old) and older children (children, 13 to 168 months old). The age of the children with PI was lower than those with SI and that of the healthy children because 57% of the patients with PI were infants (Table 1). Thus, as expected for an endemic area, PI and SI infections were particularly dominant in infants and children, respectively. As noted previously [20], frequency of detectable NS1 in plasma was higher in PI than in SI, and detection of DENV-IgM tend to be higher in SI. The infecting DENV was typeable in 66% of the cases. DENV-1 and DENV-2 were the predominant serotype in PI and SI, respectively (Table 1). Consistent with regional reports showing low circulation of DENV-4, this serotype was only detected in one case. Of note, in 7 DENV-infected infants with detectable levels of plasma DENV-specific IgG in acute phase, we were unable to establish whether SI occurred because of presence of circulating maternal IgG in infants and were not included in Table 1.

**Table 1:** Characteristics of children enrolled in the study.

		Dengue		
	Healthy (n = 40)	PI (n = 49)	SI (n = 60)	P
**Gender**				P = 0.48^a^
Female/Male	16/24	25/24	24/36	
**Age**				P<0.001^a^
6–12 months, n (%)	5 (12)	28 (57)	-	
>12 months, n (%)	35 (88)	21 (43)	60 (100)	
Age median (range)	72 (7–156)	12 (6–122)	67 (6–168)	P = 0.001^b^
**Diagnostic test**				
NS1^+^, n (%)	-	49 (100)	33 (55)	P<0.001^a^
IgM^+^, n (%)	-	46 (94)	60 (100)	P = 0.082^a^
RT-PCR+, n (%)	-	29 (60)	43 (71)	P = 0.24^a^
DENV-1, n (%)	-	21 (43)	15 (25)	P = 0.068^a^
DENV-2, n (%)	-	7 (14)	25 (41)	P = 0.003^a^
DENV-3, n (%)	-	1 (2)	2 (3)	P = 1.0^a^
DENV-4, n (%)	-	0 (0)	1 (2)	P = 1.0^a^
Non-typeable	-	20 (40)	17 (28)	P = 0.22^a^

^a^Fisher´s exact test.

^b^ Kruskal-Wallis test.

Note: Seven infants with detectable levels of plasma DENV-specific IgG in acute phase (maternally transferred or not) were not included in the Table.

### Kinetics of the response of circulating ASC in infants

**Fig 1:**
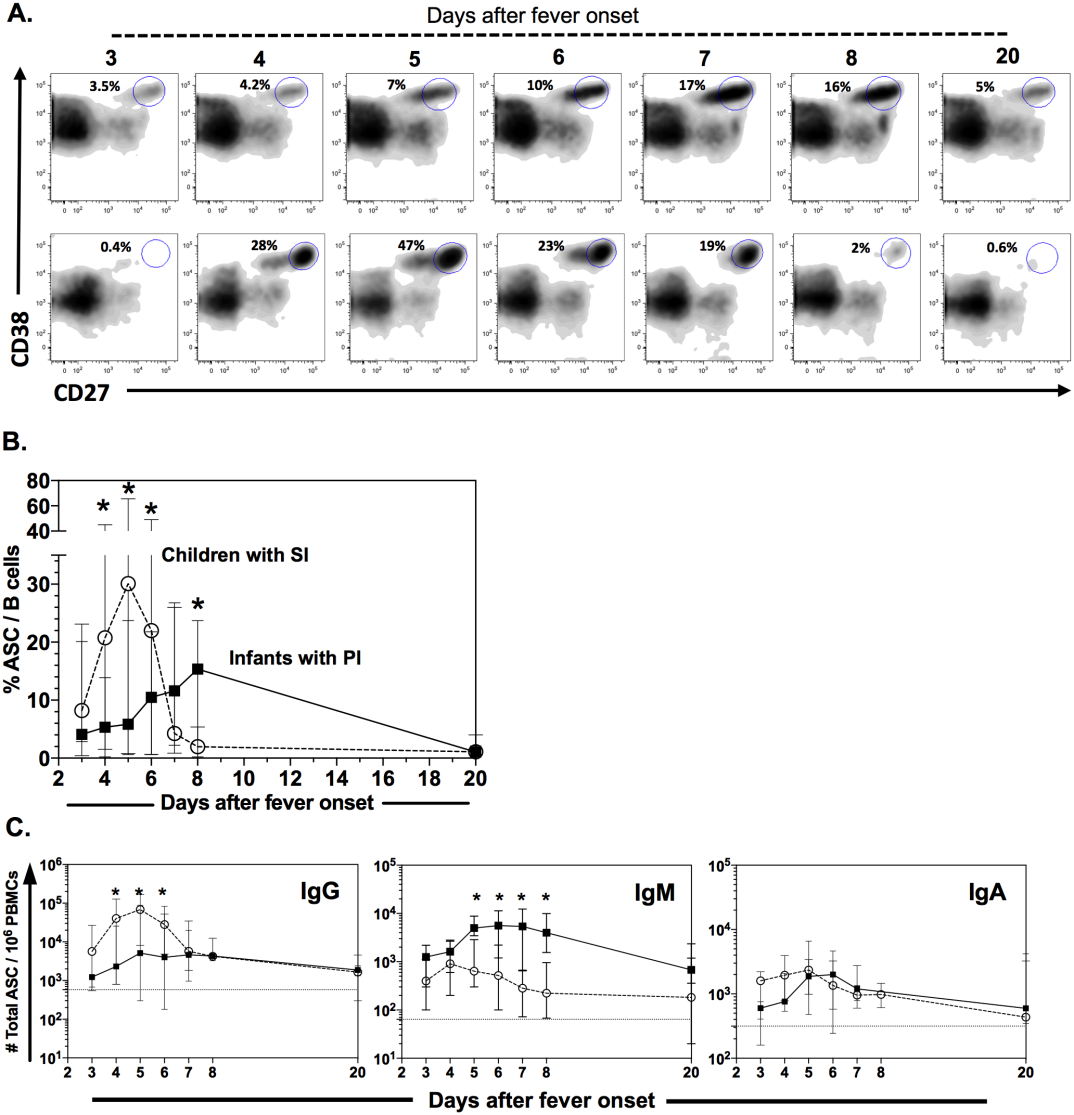
Infants with primary dengue had lower, delayed and IgM-dominated response in circulating ASC than children with SI. PBMCs were isolated from DENV-infected patients and the ASC within CD19^+^ cells was evaluated daily at acute (days 3 to 8) and convalescent phase (at day 20) by FC and two-color ELISPOT. (A) Representatives experiments showing ASC kinetic in one infant with PI (upper panels) and child with SI (lower panels). (B) Summary of the frequency of circulating ASC (median and range for each day) of 11 infants with PI (filled squares) and 15 children with SI (open circles), evaluated as shown in panel (A). (C) Frequency of ASC expressing total IgG, IgM and IgA evaluated by two-color ELISPOT in 8 infants with PI (filled squares) and 11 children with SI (open circles). Horizontal dotted line represents the median of the frequency of IgG, IgM and IgA ASC obtained from healthy controls (n = 34). * Significant differences between infants and children in the respective day (P<0.01, Mann-Whitney test).

The kinetics of circulating ASC was evaluated in a fraction of infants with PI (n = 11) and children with SI (n = 15). For this purpose, the frequency of CD19^+^CD27^hi^CD38^hi^ cells was determined by FC from day 3 to 8 after the onset of fever and in convalescence phase (Fig 1A). In the healthy children (n = 40), the median (range) value for the ASC was 0.65% (0.2–2) of circulating Bc (data not shown). A detectable ASC response was observed in the infants (Fig 1A, upper panel) and older children (Fig 1A, lower panel) during the acute phase of dengue from the third day of disease, and the response decreased significantly in convalescence. Consistent with previous reports [10, 11], in the children with SI, the response of total ASC was massive and occurred early, with a peak median (range) of 32% (1–65) for ASC/Bc on day 5 after fever onset (Fig 1B). In contrast, a 2 to 10 fold lower ASC response was detected in infants with PI on days 4, 5 and 6 after fever onset (P<0.009, Mann-Whitney test). From day 5, a gradual but steady increase in the ASC frequency was noted, with a maximum frequency on day 8 (the last day evaluated in the acute phase), with a median (range) of 15% (0.2–22) (Fig 1A and 1B).

To corroborate and extend these results, the daily dynamics of total circulating IgG, IgM and IgA ASC was also evaluated by two-color ELISPOT assays. As shown in Fig 1C, on the fifth day of fever, a massive IgG ASC response was detected in the children with SI but not in the infants with PI, who had a very slow and gradual increase in the IgG ASC frequency (Fig 1C). In contrast, IgM ASC dominated in the infants with PI, and significantly higher frequencies than those in children with SI were found on days 5, 6, 7 and 8 after symptom onset (Fig 1C). When the total IgA ASC were evaluated, a significant increase was found relative to the healthy children, but no differences in the frequency of these circulating ASC occurred between the children and infants with dengue on any of the days evaluated (Fig 1C). In the healthy children (n = 34), the median (range) for total IgG, IgM and IgA ASC/10^6^ PBMCs was 580 (136–2,374), 64 (12–460) and 315 (50–1,640), respectively. In summary, PI by dengue in infants induced a smaller and delayed response dominated by IgM-expressing ASC when compared with children with SI, in whom the response was early, rapid, massive and dominated by IgG ASC.

**Fig 2:**
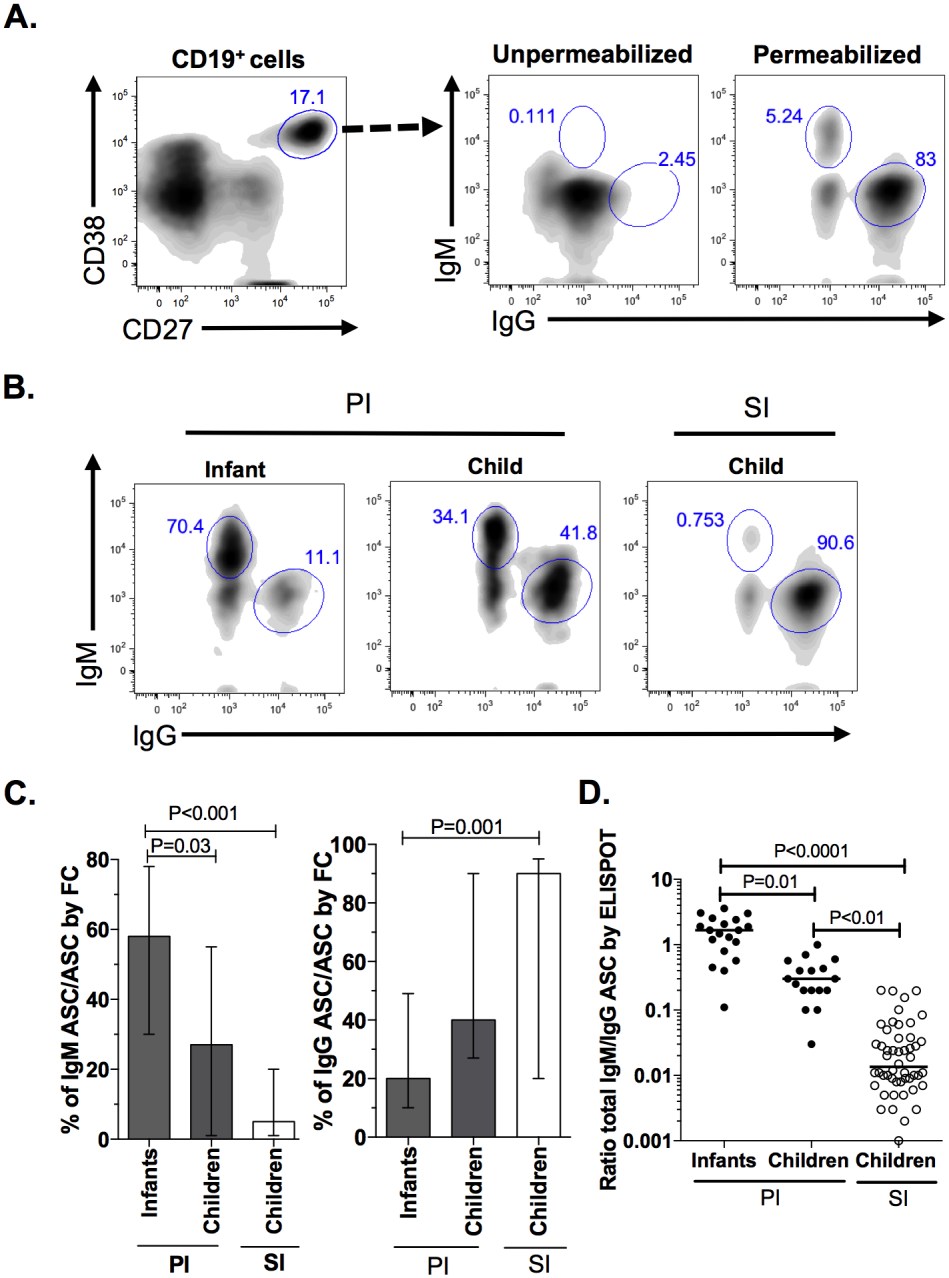
Response of total ASC induced by DENV infection is age-dependent and modulated by subsequent infections. Extra and intracellular expression of IgG and IgM in circulating ASC from infants and children with PI and children with SI were evaluated by FC. (A) FC staining was performed on un-permeabilized and permeabilized ASC from children with SI, to show predominant intracellular expression of Igs in ASC. One representative experiment of 6 is shown. (B) Intracellular staining of ASC from infants and children, both with PI, and children with SI. One representative experiment of 15 for PI and 18 for SI is shown. (C) Summary of the experiments showing in (B). Medians and ranges are presented. (D) Ratio of frequency of total IgM/IgG ASC evaluated by two-color ELISPOT in infants and children with PI and children with SI. Horizontal line represented the median. P values of post hoc test are shown.

To further determine the effect of age and pre-exposure history to the virus on the total ASC response to dengue infection, the expression of extracellular and intracellular IgM and IgG in the ASC was determined by FC from infants and children with PI, and children with SI. As expected, low ASC surface expression of both immunoglobulins was observed (Fig 2A). In contrast, when the cells were permeabilized, a much higher expression of intracellular IgM and IgG was detected, confirming that these cells were ASC (Fig 2A).

As shown (Fig 2B), when the intracellular expression of IgM and IgG was analyzed in the circulating ASC of both infants and children with PI, and children with SI, important differences were noted. The median (range) for IgM ASC/ASC in infants and children with PI and children with SI was 58% (30–78), 28% (1–55%) and 4% (0.5–18%), respectively (P = 0.03 and P<0.001 for infant PI compared with children PI and infant PI compared with children SI, respectively, by Dunn’s test). When the intracellular expression of IgG was analyzed in the three groups, the children with SI had a significantly higher frequency of ASC IgG than that in the infants with PI (P = 0.001, Dunn’s test) but not in the children with PI (P = 0.09, Dunn’s test) (Fig 2C). To confirm these results, the ratio of total IgM ASC/IgG ASC obtained by two-color ELISPOT was calculated for each group (Fig 2D). This relationship was consistently higher in the infants with PI than in the children regardless of their previous immune status, confirming that the response of circulating ASC in infants was highly dominated by IgM ASC. Fig 2D shows that among the children, a significantly higher ratio of total IgM/IgG was found for PI compared with SI, with a mean (range) of 0.4 (0.02–1) compared with 0.01 (0.001–0.1), respectively (P = 0.001, Dunn’s test). Thus, total ASC response in dengue is importantly influenced by age and previous immune status.

### The E protein-specific ASC response induced by dengue is also age dependent

**Fig 3:**
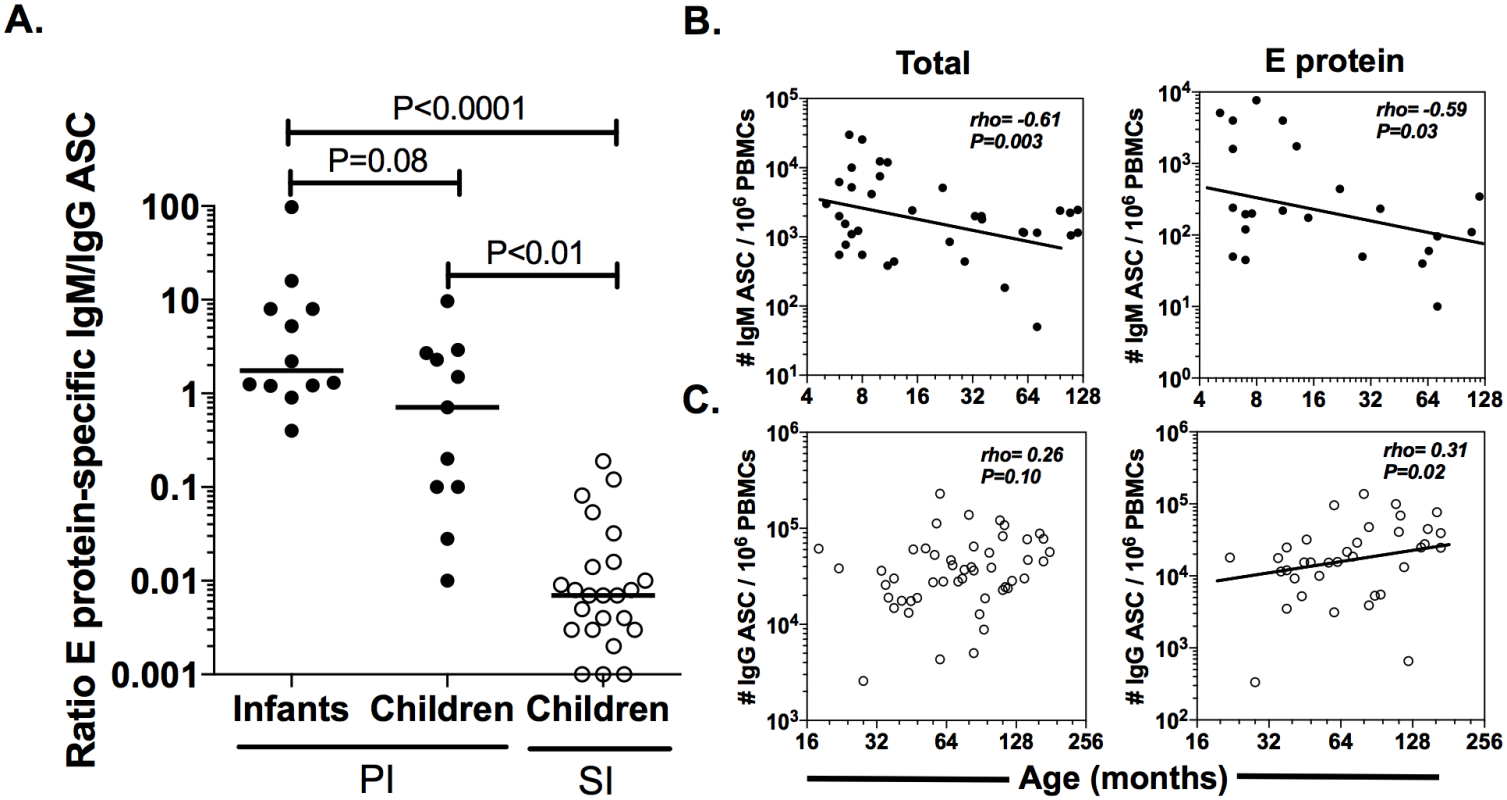
E Protein-specific ASC response is also modulated by age and subsequent infections. Frequencies of E protein-specific IgM and IgG ASC were detected by two-color ELISPOT in infants and children with PI and children with SI as control. (A). E protein-specific IgM/IgG ASC ratio is shown. (B) Frequencies of total (Left panels) and E protein-specific ASC response (Right panels) IgM and IgG ASC were plotted against age (in months) in patients with PI. (C). Same like (B) but the analysis was done on patients with SI. P value of the post hoc test, Spearman´s rho and its respective P values are shown.

To detect E protein-specific IgM and IgG ASC we developed a two-color ELISPOT assay. The median (range) of the % of E protein-specific IgG ASC over total IgG ASC was 59% (13–100) in children with SI, corroborated that the E protein is highly immunodominant [21]. Considering the results shown in Fig 2, we hypothesized that age can also modulate the virus-specific ASC response. To test this hypothesis, the E protein-specific IgM/IgG ratio was evaluated in all the groups, and the frequency of E protein-specific IgM ASC as well as IgG detected by ELISPOT was examined in relation to age. As shown in Fig 3A, and consistent with the results obtained for non antigen-specific ASC, the E protein-specific IgM/ IgG ratio was significantly higher in infants and children with PI than in children with SI. In addition, a moderate negative correlation was found in PI between age and both, total and E protein-specific ASC IgM (rho = −0.61 and rho = −0.59, respectively; P≤0.03; Spearman test). In SI, a weak positive correlation of E protein-specific IgG ASC with age was also noted (rho = 0.31, P = 0.02, Spearman test). No correlation with age was found for total or virus-specific IgG ASC in PI or total IgG and IgM ASC in SI (Fig 3C and data not shown). These results show that the age at which DENV infection occurs is a critical factor in modulating the immunoglobulin isotype of the specific response.

### IgG seropositivity modulates the response of ASC induced by dengue in infants

**Fig 4:**
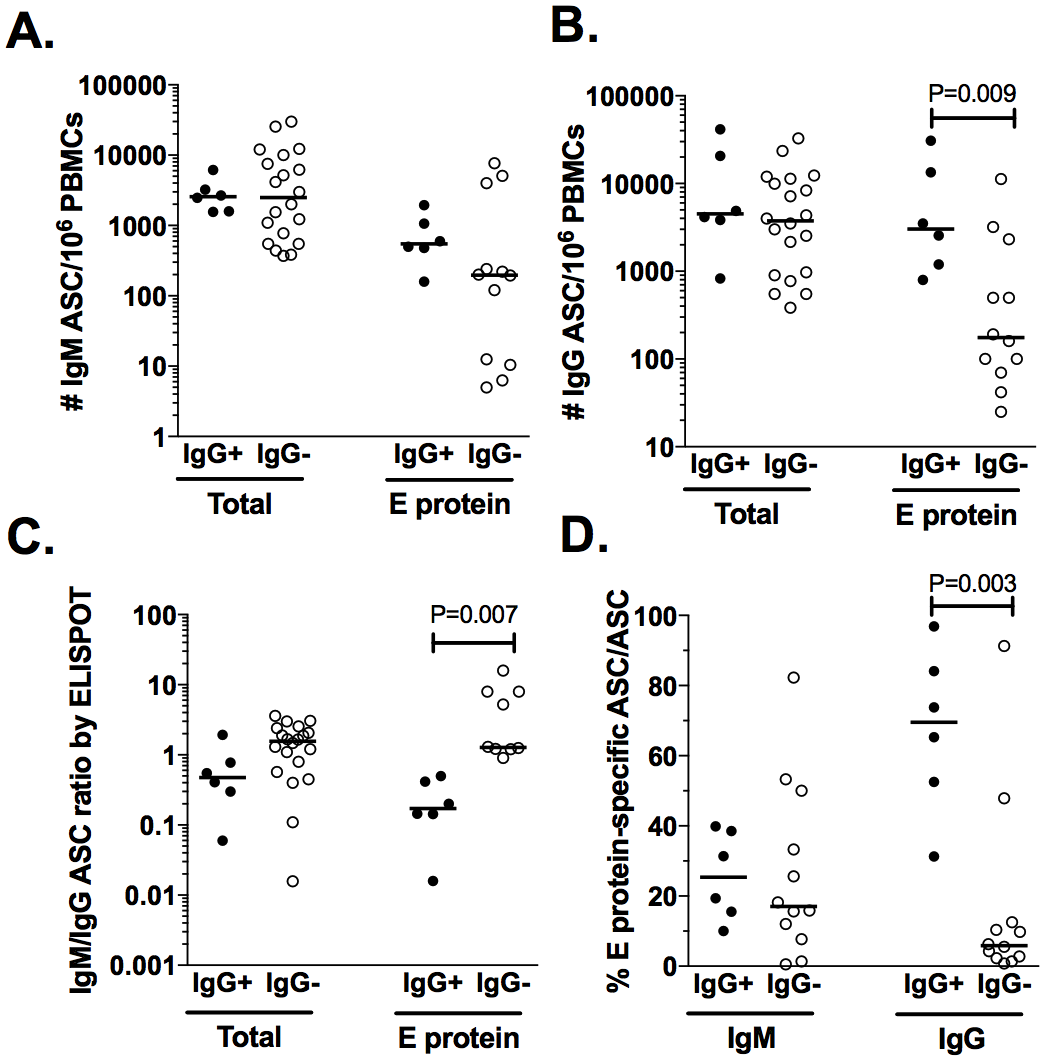
E protein-specific ASC response is differentially modulated in seropositive infants. Infants acutely infected with DENV were divided in seropositive (IgG+) and seronegative (IgG-) depending of presence o absence, respectively, of early detectable levels of DENV-specific IgG and the frequencies of total and E protein-specific IgM (A) and IgG ASC (B) were determined by two-color ELISPOT. (C) Ratios of total and E protein-specific IgM and IgG are shown. (D) Percentage of E protein-specific ASC within overall IgM and IgG ASC response. Each point represents an independent patient. Horizontal lines represent the median. The P values of Mann-Whitney test are shown.

In addition to age, IgG seropositivity (potentially from maternal origin or not) is a factor that could modulate the ASC response induced in natural DENV infections. Based on the presence of detectable plasma levels of DENV-IgG by ELISA, the infants were divided into seropositive and seronegative groups, and the frequency of total and E protein-specific IgM and IgG ASC determined by ELISPOT was evaluated in the two groups. Frequency of total and E protein-specific IgM ASC was comparable in both groups of infants (Fig 4A). The seropositive compared with the seronegative infants had a higher frequency of E protein-specific IgG ASC, with median (range) of 3,030 (800–30,720) compared with 176 (25–11,280), respectively (P = 0.009, Mann-Whitney test, Fig 4B). The seropositive infants had a significantly lower E protein-specific but not total ASC IgM/IgG ratio (Fig 4C), and the percentage of dengue-specific ASC in the total IgG ASC of the seropositive and seronegative infants was 69% (31–96) and 6% (1.4–12), respectively (P = 0.003, Mann-Whitney test) (Fig 4D). In summary, the previous existence in plasma of IgG antibodies against DENV favored the appearance of a predominantly IgG ASC virus-specific response in infants that was comparable on occasion to the response of children.

In a set of experiments (n = 7 children), the reactivity of the ASC induced by DENV infection to E protein from each of the four serotypes was tested by ELISPOT. The currently infecting serotype was identified by RT-PCR and comprised three infections by DENV-1 and two each by DENV-2 and DENV-3. As shown in S1 Fig and previously reported [11], the IgM and IgG ASC response was highly cross-reactive to E proteins of the four serotypes, and of the seven evaluated cases, only one with a primary DENV-1 infection showed any evidence of a serotype-specific response (S1 Fig).

### Kinetics of circulating Bc subpopulations in infants and children with DENV infection

**Fig 5:**
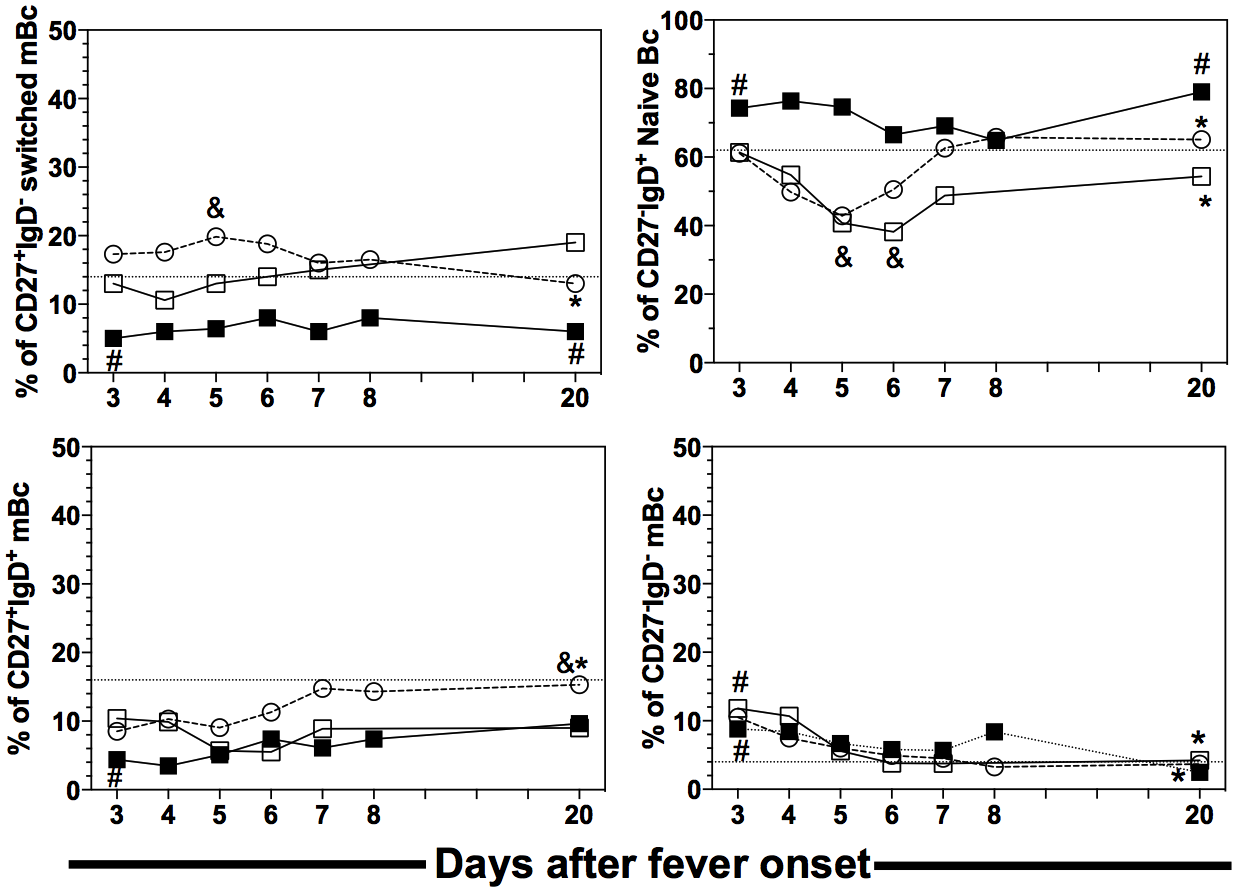
Daily dynamic of the majors circulating human Bc subsets in dengue infected patients. Frequency of switched mBc (CD27^+^IgD^-^), naïve Bc (CD27^-^IgD^+^), IgM^+^ mBc (CD27^+^IgD^+^) and CD27^-^ mBc (CD27^-^IgD^-^), were daily analyzed by FC in infants (filled square, n = 11) and children (open square, n = 5) with PI and children with SI (open circles, n = 15) in acute (from 3 to 8 day) and convalescent phase (later day 20). For simplicity purpose, only medians are shown. ^&^ Significant P values when compared with infants PI at the respective day. * Statistical significant differences when compared with the peak day in acute phase. ^#^ Significant statistical differences when compared with healthy children at the respective day. Infants and children with PI, children with SI and healthy children were compared by Kruskal-Wallis and post hoc test in each day. P<0.05 by post hoc was considered as significant. Horizontal dotted lines represent the median results of healthy children (n = 40).

In addition to circulating ASC, we analyzed the daily dynamics of the main subpopulations of total Bc: Naïve Bc (CD27^−^IgD^+^), switched memory Bc (mBc) (CD27^+^IgD^−^), IgM+ mBc (CD27^+^IgD^+^) and CD27^−^ mBc (CD27^−^IgD^−^), in a fraction of infants and children with PI and children with SI. Secondary DENV infection induced a mild but significant increase in the relative frequency of circulating Bc (data not shown) and switched Bc in the children compared with the healthy ones or those in the convalescent phase (Fig 5). The children with PI and SI had an early (starting from day 3), profound and significant decrease in naïve Bc, which were restored to normal levels in the convalescence phase. The PI infants also had slightly increased total Bc frequencies (data not shown) and generated a mild but sustained and delayed drop in the frequency of naïve Bc, which occurred later than day 6 after fever onset (Fig 5). As expected and supporting the accuracy of the cytometry assay used here, infants with PI had higher frequency of naïve Bc as well as a significantly lower frequency of switched and IgM mBc, when compared with PI or SI children in the convalescent phase (Fig 5). Interestingly, DENV infection in all the studied groups induced an early significant increase in the CD27^−^ mBc population, a difference that was lost during the convalescent phase. In short, dengue induced differential kinetic variations of the main Bc populations in the infants and children.

### Response of dengue-induced ASC is related to clinical severity in children

Using the revised WHO classification of 2009, the patients included in the study were clinically classified based on the absence or presence of dengue warning signs and the occurrence of severe infection in the following categories: DNS, DWS and SD, respectively. As shown in Table 2, marked thrombocytopenia was observed in the children with SD. The partial thromboplastin time (PTT) and aspartate aminotransferase (AST) were significantly higher in SD compared with the other classes of DENV infection (Table 2). Clinical signs, such as the magnitude of pleural effusion and increased liver size, were also significantly higher in the children with SD than in those with DWS. No significant differences occurred in the number of disease days at the moment of inclusion in the study or in any other biochemical markers, such as total creatine kinase (CK) or muscle-brain specific (CK-MB), among the children with DWS and SD (Table 2). Thus, these data confirmed the appropriate clinical classification of the analyzed patients.

**Table 2:** Clinical and laboratory characteristics of the patients with dengue included in the study.

	Dengue			
Parameter	DNS (n = 34)	DWS (n = 49)	SD (n = 33)	P
**Days of fever**	4 (2–5)	4 (2–6)	4 (2–6)	0.117^a^
**Leukocytes/μL, 1x10**^**3**^	4.2 (1.5–9.9)	7.3 (2–42)	7.5 (2.2–24)	P = 0.1^a^
**Platelets/μL, 1x10**^**3**^	144 (83–235)	56 (13–334)	42 (13–199)	<0.001^a^
**PTT, Sec**	37 (23–56)	38 (28–59)	49 (30–75)	<0.001^a^
**AST, U/L**	-	107 (30–875)	210 (58–9518)	0.0003^b^
**ALT, U/L**	-	44 (13–353)	53 (19–1603)	0.08^b^
**CK, U/L**	-	50 (24–613)	116 (28–6212)	0.11^b^
**CK-MB, U/L**	-	27 (6–42)	41 (10–183)	0.05^b^
**Pleural effusion (%)**	-	3 (0–25)	27 (0–40)	<0.001^b^
**Increased liver size, cm**	-	3 (0–6)	4 (0–7)	0.002^b^

Median (range) is shown. P of ^a^ Kruskal-Wallis and ^b^ Mann-Whitney tests are shown. DNS: Dengue non-warning signs. DWS: Dengue warning signs. SD: Severe dengue. PTT: Partial thromboplastin time. AST: Aspartate aminotransferase. ALT: Alanine aminotransferase. CK-MB: Creatine kinase muscle-brain specific.

**Fig 6:**
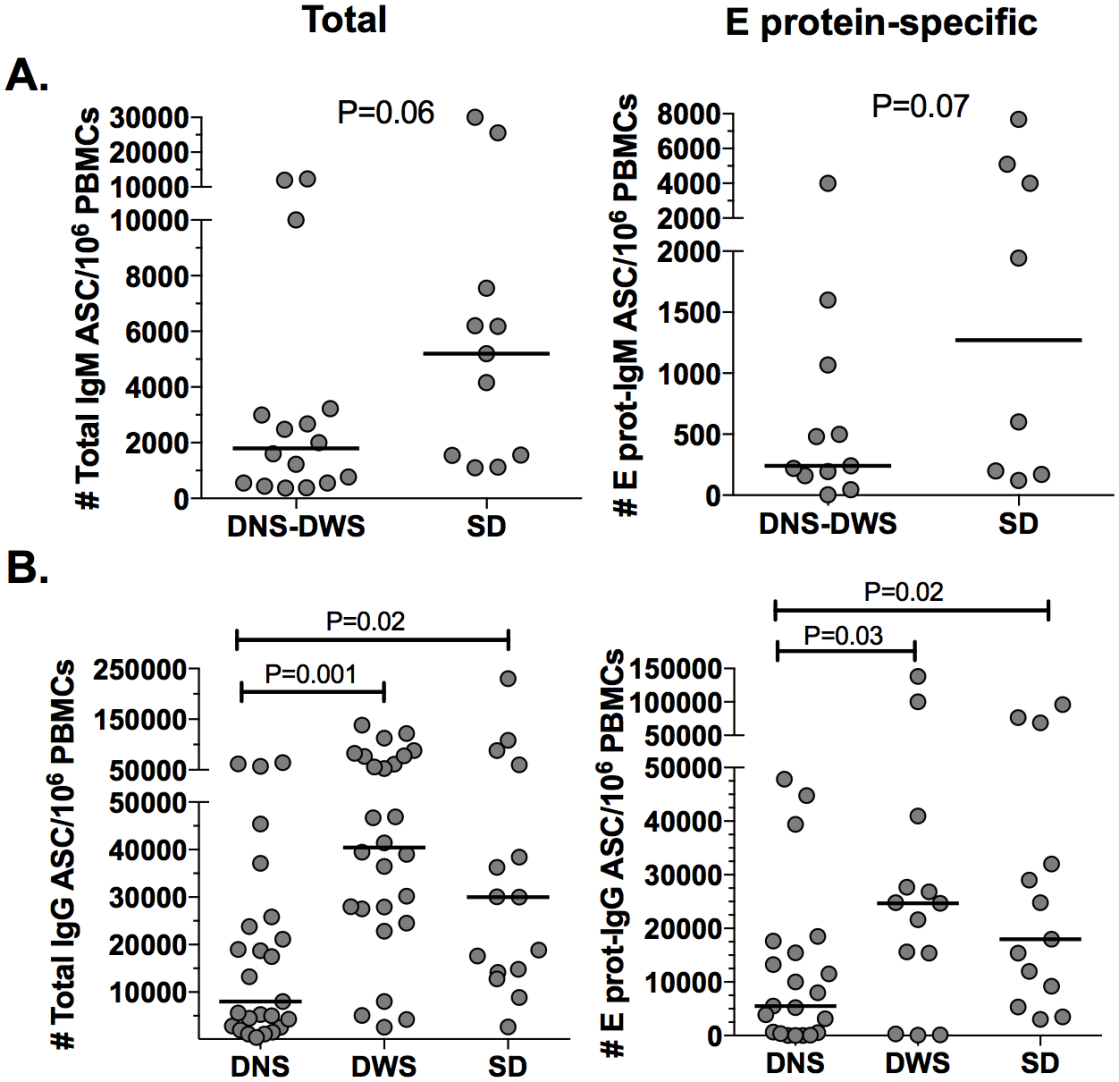
Response of circulating ASC is related with the clinical outcome. Frequencies of total and E protein-specific IgM and IgG ASC detected by two-color ELISPOT were analyzed in patients with dengue clinically ranging from mild to severe, using the 2009-revised WHO classification. (A) Total and E protein-specific ASC response in infants and (B) children is shown. Horizontal line represented the median. Each point corresponds to one independent experiment. P values of the Mann-Whitney (A) and post hoc test (B) are shown. DNS: Dengue no-warning signs. DWS: Dengue with warning signs. SD: Severe dengue.

The response of total and E protein-specific ASC was evaluated in each of the clinically classified groups. Because age is a key factor that modulates the ASC response, the patients here were also divided into infants and children. Of note, clinically, the infants were classified as having dengue (DNS plus DWS) or SD because of the few infants with PI carrying the DNS diagnosis. The frequency of total IgM ASC/10^6^ PBMCs in the infants with dengue compared with severe form of dengue was 1,800 (370–12,000) and 5,200 (1,100–30,000), respectively (P = 0.06, Mann-Whitney test) (Fig 6A); the frequency of E protein-specific IgM ASC/10^6^ PBMCs was 240 (5–4,000) and 1,271 (120–7,680), respectively (P = 0.07, Mann-Whitney test) (Fig 6A), showing a tendency for higher elevation of total and specific IgM ASC in children with SD. There were no significant differences for total or specific IgG ASC in infants (data not shown).

A 3- to 6-fold (for total) and 3- to 5-fold (for E protein) significant higher frequency of IgG ASC was found in the children with more clinically relevant forms of infection (DWS and SD) compared with those with DNS (Fig 6B). When only children with SI were analyzed, this difference was even greater (P = 0.009, DNS compared with SD, Mann-Whitney test, data not shown). Thus, the magnitude of the IgM ASC response in infants and of the IgG ASC response in older children was related to the clinical form of the disease.

### Relation of the E protein-specific ASC response to the respective titers of plasma Igs

Finally, we analyzed the relationship between the frequencies of E protein-specific IgM and IgG ASC and the respective titers of plasma Igs from acute samples. Plasma E protein-specific IgG was efficiently evaluated by ELISA. However, this was not technically possible for IgM (data not shown), and the relative concentration of IgM was evaluated against the complete virus, by testing serial dilutions of plasma on a Panbio capture ELISA commercial kit.

**Fig 7:**
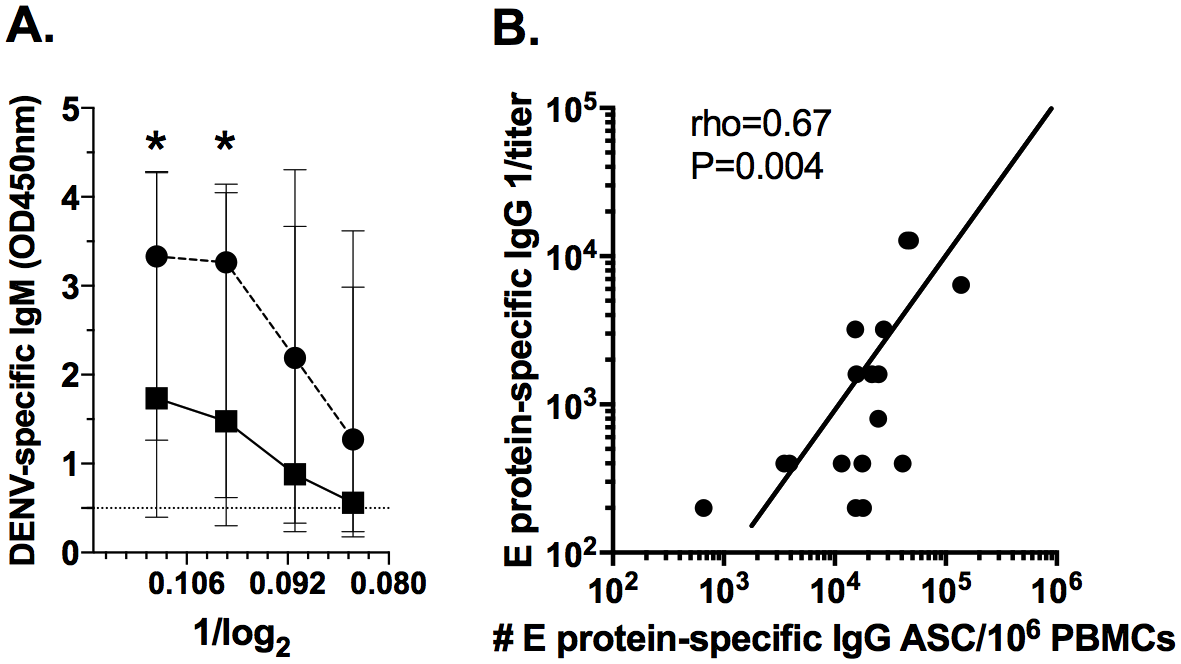
Relations between frequencies of virus-specific ASC and the respective Igs plasma titers. (A) The relative amount of DENV-specific IgM (against whole DENV) in plasma was evaluated by ELISA in infants (circles, n = 17) and children (squares, n = 16) with PI using a commercially available kit (DENV-IgM capture, Panbio) and serial dilutions. Median and ranges of the obtained optical density at 450nm (OD450nm) are shown. (B) The correlation between the frequencies of E protein-specific IgG ASC analyzed by ELISPOT and the respective titers of plasma IgG evaluated by ELISA in children with SI (n = 20). * Significant differences by Mann-Whitney test. The rho and P of Spearman rank test are shown.

In PI the relative amount of DENV-specific IgM was significantly higher in infants than in children (Fig 7A). For IgG, a moderate significant positive correlation between the frequency of E protein-specific ASC and the titer of respective plasma Igs in children with SI was found (rho = 0.67, P = 0.004, Spearman test). These results support the hypothesis that during the acute phase of DENV infection in children, virus-specific Igs in plasma could reflect, at least partly, the magnitude of the response of respective circulating ASC.

## Discussion

In this study, we analyzed the dynamics, reactivity and association with clinical outcome of naturally induced ASC in children with primary and secondary DENV infection. Because a significant fraction of the patients were infants, the influence of age on the ASC response could also evaluated.

As noted in previous studies of adults and older children with subsequent infections [9–11], dengue induced a substantial increase in the frequency of the ASC that circulate in the acute phase (Fig 1A). Daily detailed evaluations showed that the response in children with SI was early and rapid, peaking on the fifth day after symptom onset (Fig 1B). In general, regardless of age or disease type, the ASC response to dengue was shown to be especially intense and self-limiting, distinct from findings for other viruses such as respiratory syncytial virus, for which virus-specific ASC have been detected up to 20 days after symptom onset [22]. Factors that favor the development, survival or differentiation of Bc, particularly cytokines and certain members of the TNF superfamily, have been evaluated in dengue. *In vitro* infected human endothelial cells and monocytes of patients with dengue secrete detectable amounts of BAFF and APRIL, respectively [23, 24]. A particular subpopulation of monocytes expressing CD16 selectively expanded in dengue, favoring the terminal differentiation of Bc through the production of IL-10 [25], a cytokine regularly increased in this viral infection.

Unlike the situation in SI in children, the infants with PI generated a slow, delayed and lower-intensity response dominated particularly by IgM ASC (Fig 1B and 1C). Various acute viral infections such as hepatitis A and rotavirus are also characterized by an increase in circulating IgM ASC [26, 27]. The total ASC response differed between the infants and children, both with PI (Fig 2B and 2C), confirming that the immune response early in life is distinct. For example, dendritic cells of infants express low levels of MHC II and costimulatory molecules and secrete less IL-12p70 compared with those of adults [28, 29]. Differences in age-related innate immune responses have also been seen in dengue. Purified monocytes from healthy infants infected with DENV *in vitro* produce significantly less nitric oxide and proinflammatory cytokines compared to monocytes from adults [14, 30]. Infants show an impaired humoral response to capsular bacterial antigens; thus, the Bc response in infants is also distinct. The naïve Bc of infants showed a decreased expression of CD21, CD80 and CD86, molecules that mediate Bc activation [31]. This altered expression of costimulatory molecules could be related with the lower magnitude of the ASC response found in the infants infected with DENV, compared with children (Fig 1).

The acute phase ASC response was dominated by total and DENV-specific IgG ASC in the children, regardless of the type of infection, but not in the infants with PI, in which IgM ASC predominated (Fig 3A), suggesting that in infants infected with DENV, ASC could have an impaired ability to switch to the IgG isotype. The infants Bc show a decreased CD40 expression, a critical molecule involving in the isotype switching [31]. This abnormality is diminished with increasing age, given that an early detectable response of DENV-specific IgG ASC in adults with PI has been reported [32]. Consistently, a negative correlation was found between age and the capacity to produce total and DENV-specific IgM (Fig 3B). For SI, a greater capacity to produce DENV-specific IgG was acquired over time (Fig 3C); thus, age is a critical factor in the immunity to dengue. In PI and SI the ASC response evaluated in acute phase was highly cross-reactive between four serotypes (S1 Fig). A limitation for the present study is that the long-term reactivity of the ASC response or plasma, where a type-specific response is expected, was not evaluated.

Compared with the seronegative, the infants seropositive for DENV-IgG in acute phase had a higher IgG ASC response, and the response was dominated by E protein-specific ASC, some time comparable to children with SI (Fig 4). We were unable to determine whether these infants had PI (with the presence of maternally transferred Igs) or suffered from SI. Dengue-specific maternal Igs through antibody-dependent enhancement could increase the magnitude of ASC response and favoring the isotype switching to IgG. However, although transferred Igs can be detected for up to 12 months of age, around of 30% of infants maintain detectable levels of maternal DENV-specific IgG in plasma at 6 months [33], the minimum age of inclusion for the infants analyzed here. After priming, infants are predisposed to differentiate of Bc toward mBc rather than plasma cells, so early immunization may fail to induce a detectable antibody response [34]. However, in a subsequent event, a strong and massive antigen-specific response, as noted here, can be detected [34]. These results have important implications for dengue vaccines because the ideal age for vaccination is currently in discussion.

Dengue infection also caused disturbance in the frequency of several populations of circulating Bc, with a drastic decrease in the frequency of naïve Bc (Fig 5). This decrease was closely related to the expansion of the circulating ASC population, so this change in the distribution of Bc populations in SI is potentially due to a replacement of one subpopulation by another [9]. Naïve Bc and mBc have differential capacity to produce cytokines and antigen presentation [35]; thus, this change in the distribution of Bc subpopulations could influence, at least temporarily, disease pathogenesis, for example the pattern of plasma cytokines, as noted in patients with rheumatoid arthritis treated with anti-CD20 depletion therapy [36]. Dengue induced the acute expansion of CD27^−^ mBc (Fig 5), a finding similar to that noted in respiratory syncytial virus [37], malaria y HIV infections.

A trend toward higher frequencies of total and E protein-specific IgM ASC circulating in infants severely affected by dengue was observed (Fig 6A). Clinically relevant cases are presented more frequently in patients with than without detectable plasma DENV-IgM [38]. However, high levels of IgM were also associated with low viral load [39] and a significant remaining early neutralizing activity against DENV is due to IgM [32], supporting the protective role of DENV-IgM. For primary infections, higher plasma levels of DENV-specific IgM occurred in infants than in children (Fig 7B); thus, the immunity against DENV mediated by IgM for example through complement cascade activation could be relevant to the early age response. In children, regardless of the type of infection, a higher DENV-IgG, but not IgM, ASC response was found in clinically severe cases (Fig 6B). Our results confirming and extending previous reports in which adults with DENV infections were analyzed, and found that the same difference of greater magnitude [9]. Why the ASC response is associated with clinical severity is unknown. However, ASC can produce cytokines [40] previously related with dengue pathogenesis. Recently we demonstrated that activated B cells (possibly plasmablasts) isolated from children with dengue release soluble CD27, and the plasma level of this antigen is elevated in children with SD [41]. Thus, the response of total and virus-specific ASC could be a new biomarker associated with severity in pediatric settings.

Acutely circulating ASC appear to be partially responsible for the E protein-specific IgG titers found during the acute phase of SI (Fig 7B) [10]. In summary, DENV infection induces a strong ASC response that is highly modulated by age and subsequent infections and is related with the clinical outcome. These findings are important for the development of dengue vaccines.

## Supporting Information

S1 Fig Response of ASC is highly cross-reactive between four DENV serotypes. ASC obtained from acutely infected children were tested against recombinant E protein from each of four DENV serotypes or a mix of all (to obtain the whole response) by two-color ELISPOT. Currently infecting serotype was identified by RT-PCR. (A) The percentage (in bars) of E-protein-specific IgM and (B) IgG ASC of 7 patients (3 PI and 4 SI) reacting with every serotype is shown. Arrows indicate one patient (with PI) with IgM and IgG serotype-specific ASC response.
(TIFF)
