# Supplementary figures and images for: Total and Envelope Protein-Specific Antibody-Secreting Cell Response in Pediatric Dengue Is Highly Modulated by Age and Subsequent Infections
Source: PLoS One. 2016 Aug 25;11(8):e0161795. doi: 10.1371/journal.pone.0161795 (PMC4999220; doi:10.1371/journal.pone.0161795)

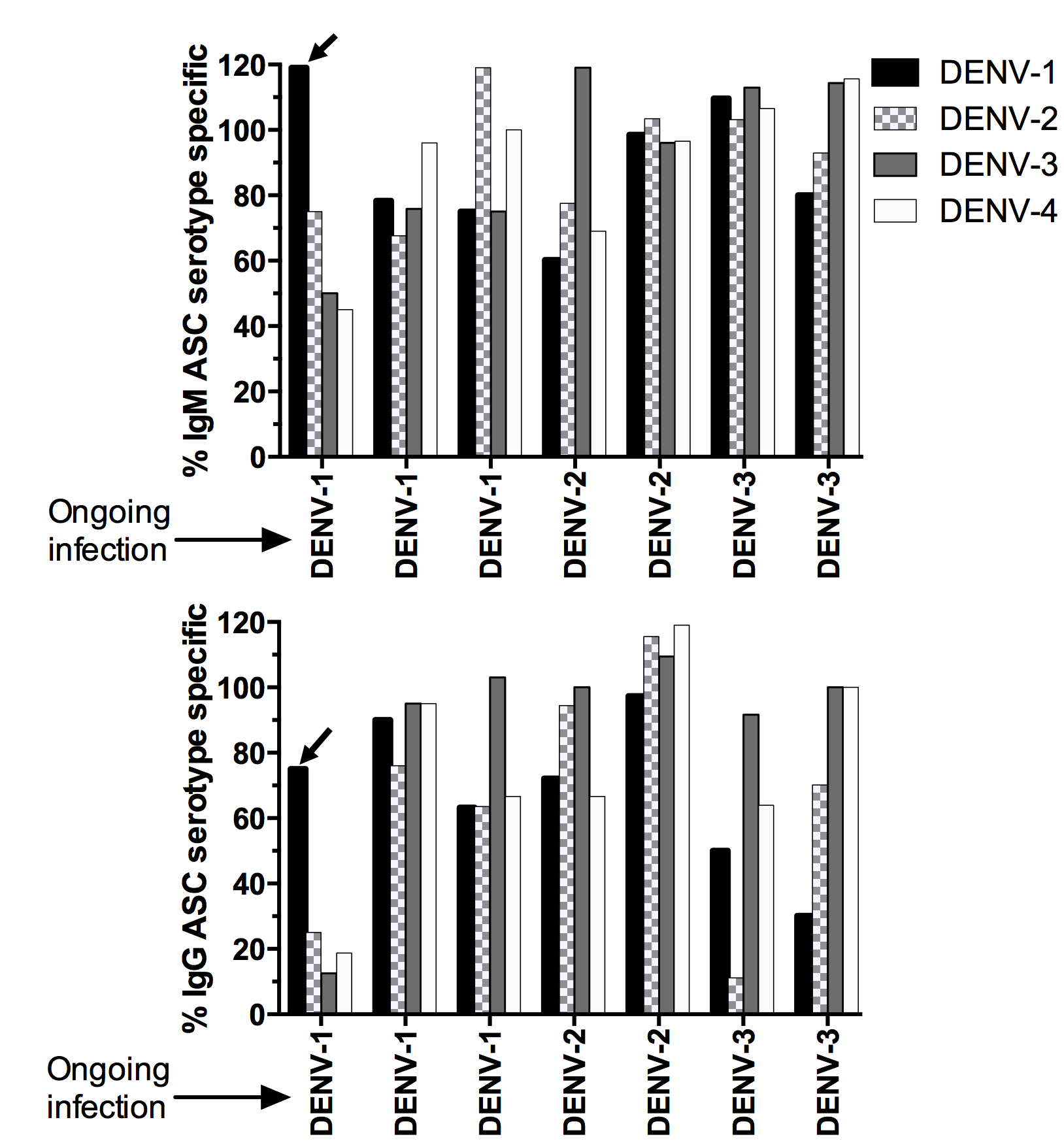

Supplement: S1 Fig — ASC obtained from acutely infected children were tested against recombinant E protein from each of four DENV serotypes or a mix of all (to obtain the whole response) by two-color ELISPOT. Currently infecting serotype was identified by RT-PCR. (A) The percentage (in bars) of E-protein-specific IgM and (B) IgG ASC of 7 patients (3 PI and 4 SI) reacting with every serotype is shown. Arrows indicate one patient (with PI) with IgM and IgG serotype-specific ASC response. (TIFF) [file pone.0161795.s001.tiff]
